# Complete Genome Sequence *of* Burkholderia vietnamiensis LMG16232

**DOI:** 10.1128/mra.00328-23

**Published:** 2023-07-03

**Authors:** Zhong Ling Yap, Anna Motnenko, Silvia Cardona

**Affiliations:** a Department of Microbiology, University of Manitoba, Winnipeg, Manitoba, Canada; The University of Arizona

## Abstract

We report here the complete annotated closed genome sequence of Burkholderia vietnamiensis LMG16232. There were three contigs, with a combined size of 6,739,172 bp and a GC content of 67%.

## ANNOUNCEMENT

Burkholderia vietnamiensis LMG16232 (CCUG 31370) was obtained in 1993 from a sputum sample from a 20-year-old cystic fibrosis patient in Göteborg, Sweden, and belongs to the Burkholderia cepacia complex strain panel, which was created for diagnostic purposes (1, 2). The strain is deposited at the Belgium Coordinated Collection of Microorganisms (BCCM) (https://bccm.belspo.be/catalogues/lmg-strain-details?NUM=16232).

The strain was obtained from the BCCM in 1999 and stored at −80°C in LB medium containing 20% glycerol. After the original acquisition, the strain was passaged three times by collecting bacterial lawns and isolated colonies from LB agar petri dishes, prior to storage. To extract the DNA, a test tube containing 5 mL of LB medium was inoculated with a loopful of the last stored stock, and the culture was incubated for 16 h at 30°C. The DNA was extracted using the PureLink microbiome DNA purification kit (Invitrogen) according to the manufacturer's instruction. Oxford Nanopore Technologies (ONT) and Illumina libraries were prepared from the same DNA preparation at the Microbial Genome Sequencing Center (MiGS) (Pittsburgh, PA) using the workflow described elsewhere (3). Briefly, the libraries for Illumina sequencing and ONT sequencing were prepared using the Illumina Nextera kit and the SQK-LSK109 kit, respectively, according to the manufacturer’s specifications; the libraries were sequenced on an Illumina NextSeq 550 platform (paired-end sequencing) and an ONT MinION system with an R9 flow cell, respectively. All reads were provided as FASTQ files (Illumina, 2,654,441 reads [698,867,643 bases, with 106× coverage]; ONT, 99,965 reads [1,304,948,489 bases, with 193× coverage and an *N*_50_ value of 13,049 bp]). Genome assembly was performed with the Galaxy bioinformatics platform (https://usegalaxy.org), using both Illumina and ONT reads. Quality control and adapter trimming were performed using FastQC (Galaxy v0.73+galaxy0) and Trimmomatic (Galaxy v0.38.1) (4) for Illumina paired-end reads. The ONT scaffold was assembled using Flye (Galaxy v2.9+galaxy0) (5). The trimmed Illumina paired-end reads were mapped to the ONT genome assembly using BWA-MEM (Galaxy v0.7.17.2) (6), and the resulting BAM file was used to polish the ONT assembly with Pilon (Galaxy v1.20.1) (7). Genome annotation was performed using Prokka (Galaxy v1.14.6+galaxy1) (8). Default parameters were used for all software unless otherwise specified.

The B. vietnamiensis LMG16232 genome was assembled into three circular replicons, with 3,314,184 bp (GC content, 67.0%), 2,219,983 bp (GC content, 67.6%), and 1,205,005 bp (GC content, 67.0%). The total size is 6,739,172 bp, and the average GC content is 67%. The coverage values for the Illumina and ONT sequences are 106× and 193×, respectively. Replicon 1 contains 3,003 coding genes, 12 rRNAs, 65 tRNAs, and 1 transfer-messenger RNA (tmRNA), replicon 2 contains 1,874 coding genes, 3 rRNAs, and 11 tRNAs, and replicon 3 contains 1,014 coding genes, 3 rRNAs, and 4 tRNAs. In total, the LMG15232 genome is predicted to have 5,891 coding genes, 18 rRNAs, 80 tRNAs, and 1 tmRNA. Compared to the previously sequenced draft genome sequenced with an Illumina sequencer (ENA accession number ERS785077), this closed genome has additional 67,835 bp.

### Data availability.

The genome sequence of B. vietnamiensis LMG16232 is available in GenBank under accession numbers CP120730 to CP120732. The raw sequence reads are available under Sequence Read Archive (SRA) accession numbers SRR24175964 (Illumina) and SRR24175963 (ONT).
